# Crystal structure and Hirshfeld surface analysis of *N*-[(2-hy­droxy­naphthalen-1-yl)(3-methyl­phen­yl)meth­yl]acetamide

**DOI:** 10.1107/S2056989018008423

**Published:** 2018-06-21

**Authors:** Khawla Boudebbous, Wissem Zemamouche, Abdelmadjid Debache, Noudjoud Hamdouni, Ali Boudjada

**Affiliations:** a Laboratoire de Synthèse de Molécules, d’Intérêts Biologiques, Département de Chimie, Université Mentouri-Constantine, 25000 Constantine, Algeria; b Laboratoire de Cristallographie, Département de Physique, Université Mentouri-Constantine, 25000 Constantine, Algeria

**Keywords:** 1-amino­alkyl-2-naphthols, crystal structure, hydrogen bonding, Hirshfeld surface

## Abstract

The title compound, C_20_H_19_NO_2_, is of inter­est as a precursor to biologically active substituted quinolines and related compounds. This compound crystallizes with two independent mol­ecules (*A* and *B*) in the asymmetric unit. The dihedral angles between mean planes of the methyl­phenyl ring and the naphthalene ring system are 78.32 (6) and 84.70 (6)° in mol­ecules *A* and *B*, respectively. In the crystal, the anti­ferroelectric packing of mol­ecules *A* and *B* is of an *ABBAABB* type along the *b*-axis direction. The crystal structure features N—H⋯O, O—H⋯O and weak C—H⋯O hydrogen bonds, which link the mol­ecules into infinite chains propagating along the *b*-axis direction.

## Abstract 2

This compound crystallizes with two independent mol­ecules (*A* and *B*) in the asymmetric unit. In the crystal, the *A* and *B* mol­ecules stack head-to-tail in columns along the *b*-axis direction.

## Chemical context   

1-Amino­alkyl-2-naphthols are used as bradycardiac (Dingermann *et al.*, 2004 ▸) and hypotensive agents (Shen *et al.*, 1999 ▸). In addition, 1,3-oxazines possess pharmaceutical properties such as analgesic (Lesher *et al.*, 1955 ▸), anti­tumor (Remillard *et al.*, 1975 ▸), anti­malaria (Ren *et al.*, 2001 ▸) and anti­biotic (Haneishi *et al.*, 1971 ▸). The above compounds are easily prepared from 1-amino­alkyl-2-naphthols (Damodiran *et al.*, 2009 ▸) and for this reason they are of great inter­est. The usual method for the preparation of 1-amino­alkyl-2-naphthols is a three-component reaction between 2-naphthol, aromatic aldehydes and acetamide catalysed by various catalysts (Singh *et al.*, 2015 ▸). For our part we propose a new method using phenyl­boronic acid as catalyst in a free-solvent medium.
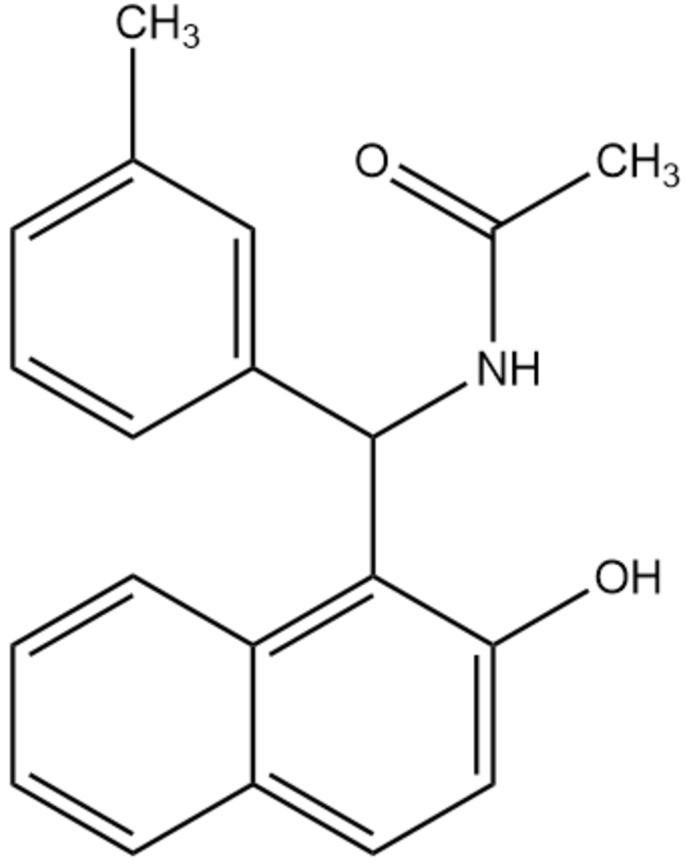


## Structural commentary   

**Figure 1:**
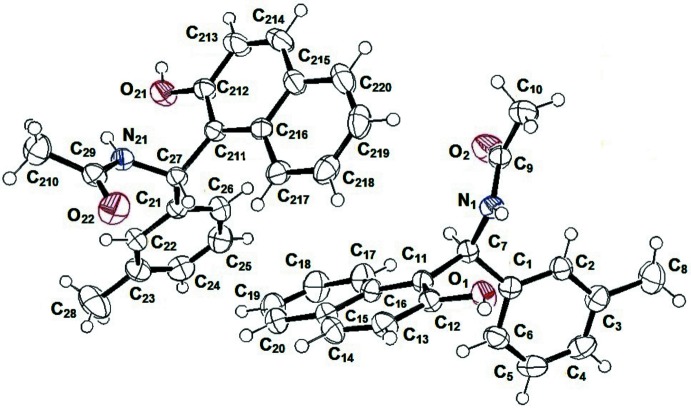
The mol­ecular structure of the title compound, with atom labelling and displacement ellipsoids drawn at the 50% probability level.

The mol­ecular structure of the title compound is shown in Fig. 1 ▸. It crystallizes with two independent mol­ecules (*A* and *B*) in the asymmetric unit, with *Z* = 8. The bond lengths in the methyl­phenyl rings and naphthalene ring systems of the two mol­ecules are practically equal, while there are slight differences in bond angles, with for example N1—C7—C1 and N21—C27—C21 differing by 1.2° and the exocyclic angles C7—C11—C12 and C27—C211—C212 differing by 1.8°. The naphthalene ring systems are essentially planar with maximum deviations from the mean plane of 0.059 (1) Å (for C11) and −0.020 (1) and 0.020 (2) Å (for C211 and C213) in mol­ecules *A* and *B*, respectively. The mean plane of the naphthalene ring system subtends a dihedral of angle of 78.32 (6)° with the methyl­phenyl ring in mol­ecule *A* and 84.70 (6)° in *B* while the dihedral angles between the naphthalene ring system and the acetamide group is 55.98 (9)° in mol­ecule *A* and 65.30 (9)° in *B*. This differences also exist between the mean plane of acetamide and phenyl rings which are about 80.63 (10)° for mol­ecule A and 84.51 (10)° for mol­ecule *B*. The methyl groups at C8 and C28 have a C–H bond eclipsed in the mean plane of the phenyl ring and they are oriented towards the acetamide group, as been observed in *N*-[(2-hy­droxy­naphthalen-1-yl)(4-methyl­phen­yl)meth­yl]acetamide (Khanapure *et al.*, 2015 ▸).

Intra­molecular N—H⋯O hydrogen bonds (Table 1 ▸) involving the hydroxyl O atoms result in the formation of pseudo six-membered rings in both mol­ecules.

## Supra­molecular features   

**Figure 2:**
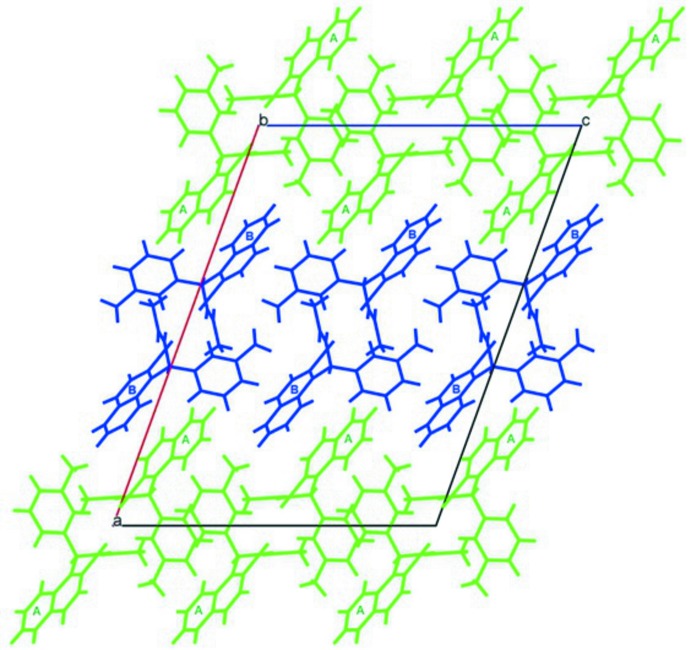
A view along the *b* axis of the crystal packing of the title compound.

In the crystal, the anti-ferroelectric packing of mol­ecules *A* and *B* is of an *ABBAABB* type (Fig. 2 ▸). Inversion-related mol­ecules are lined by pairs of hydrogen bonds (Table 1 ▸), forming infinite chains along the *b*-axis direction. O—H⋯C and C—O⋯O short contacts are also present in the crystal (Table 2 ▸).

## Analysis of the Hirshfeld surfaces   

**Figure 3:**
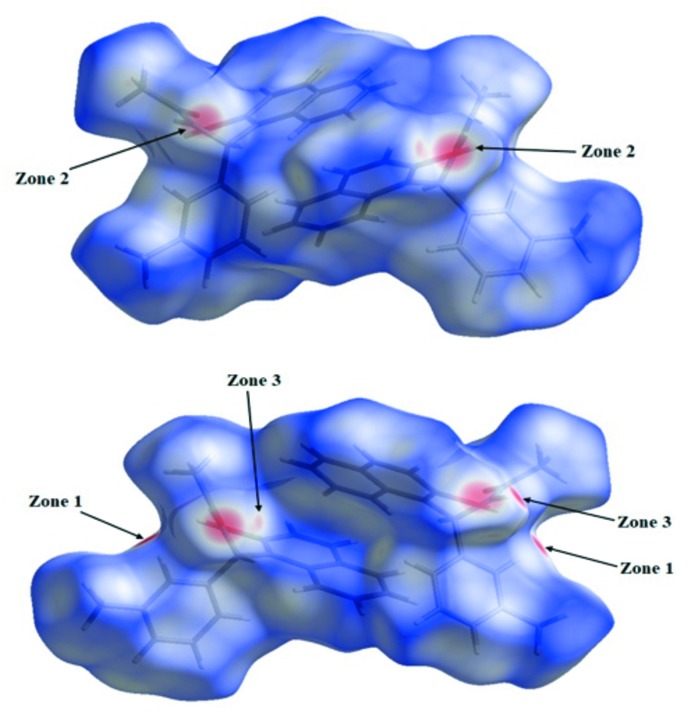
Two views of the Hirshfeld surface mapped over *d*_norm_.

**Figure 4:**
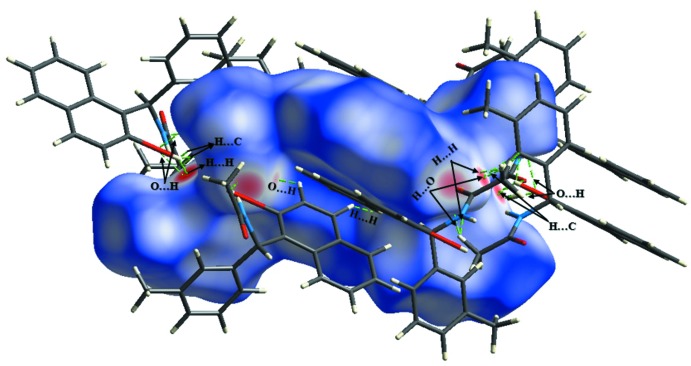
A view of the Hirshfeld surface mapped over *d*_norm_, with neighbouring inter­actions shown as green dashed lines.

**Figure 5:**
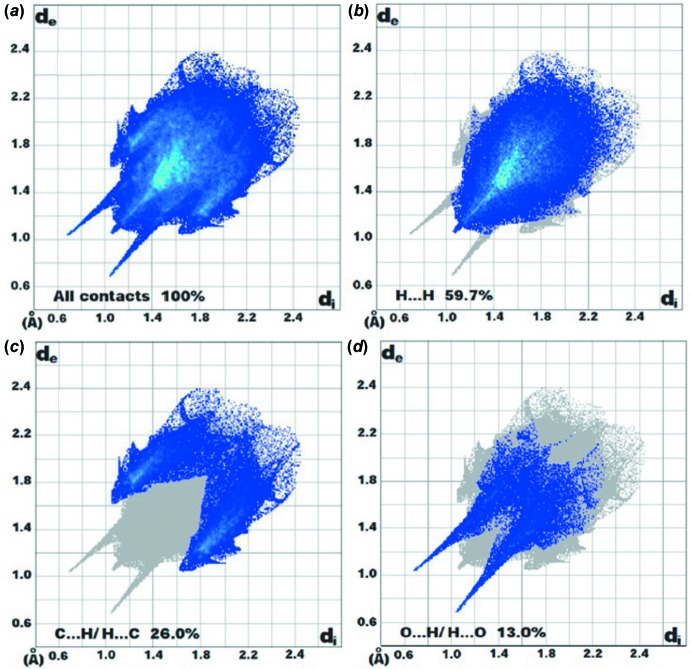
Two-dimensional fingerprint plots: (*a*) overall, and delineated into contributions from different contacts: (*b*) H⋯H, (*c*) H⋯C/C⋯H and (*d*) H⋯O/O⋯H.

The Hirshfeld surface analysis (Spackman & Jayatilaka, 2009 ▸) and the associated two-dimensional fingerprint plots (McKinnon *et al.*, 2007 ▸) were generated with *CrystalExplorer* 3.1 (Turner *et al.*, 2017 ▸). The Hirshfeld surface of the compound mapped over *d*_norm_ is illustrated in Fig. 3 ▸. The red spots in Fig. 4 ▸ correspond to close H⋯H contacts resulting from the short O—H⋯H contacts, and the white areas, representing distances between neighboring atoms close to the sum of the van der waals radii, indicate N⋯H/H⋯N inter­actions. Bluish areas illustrate areas where neighboring atoms are too far apart to inter­act with one another. Fig. 5 ▸ *a* illustrates the two-dimensional fingerprint of all the contacts contributing to the Hirshfeld surface. The two-dimensional fingerprint of the points *d*_i_, *d*_e_ (Fig. 5 ▸ *b*) associated with hydrogen atoms is characterized by an extremity pointed to the origin along the *a* diagonal, which corresponds to *d*_i_ + *d*_e_ = 2.2 Å and represents 59.7% of all the inter­molecular contacts. Fig. 5 ▸ *c* illustrates C⋯H/H⋯C contacts between carbon and hydrogen atoms from inside and outside the Hirshfeld surface and *vice versa*, resulting from H⋯C short contacts. It accounts for 26.0% of the surface and is characterized by two symmetrical points with *d*_i_ + *d*_e_ = 2.6 Å. The plot of O⋯H/H⋯O contacts between hydrogen atoms located inside the Hirshfeld surface and oxygen from outside and *vice versa* is shown in Fig. 5 ▸ *d*. These contacts account for 13.0% and are characterized by two symmetrical peaks with *d*_i_ + *d*_e_ = 1.8 Å; this reveals the presence of strong O⋯H contacts that are characteristic of C—H⋯O and O—H⋯O hydrogen bonds.

## Database survey   

A search of the Cambridge Structural Database (Version 5.37, update May 2016; Groom *et al.*, 2016 ▸) for *N*-[(2-hy­droxy­naphthalen-1-yl)(*m*-tol­yl)meth­yl]acetamide yielded four hits: methyl *N*-[(2-hy­droxy­naphthalen-1-yl)(phen­yl)meth­yl]carb­amate (Bazgir *et al.*, 2006 ▸), *N*-[(2-hy­droxy­naphthalen-1-yl)(phen­yl)meth­yl]acetamide (Mosslemin *et al.*, 2007 ▸), *N*-[(2-hy­droxy­naphthalen-1-yl)(4-methyl­phen­yl)meth­yl]acetamide (Khanapure *et al.*, 2015 ▸) and *N*-[(2-hy­droxy-1-naphth­yl)(3-nitro­phen­yl)meth­yl]acetamide (NizamMohideen *et al.*, 2009 ▸). Three of these compounds involve *N*-[(2-hydroxynaphthalen-1-yl) (Bazgir *et al.*, 2006 ▸; Mosslemin *et al.*, 2007 ▸; Khanapure *et al.*, 2015 ▸); in these analogues, the naphthalene ring system is inclined to the benzene ring by 81.54, 82.10 and 82.50° respectively, but in the hy­droxy-1-naphthyl compound (NizamMohideen *et al.*, 2009 ▸), the dihedral angle is 81.9°, compared with 78.32 (6) and 84.70 (6)° in mol­ecules *A* and *B* of the title compound. In the four compounds above, as in the title compound, intra­molecular N—H⋯O and inter­molecular O—H⋯O hydrogen bonds are observed.

## Synthesis and crystallization   

A mixture of *m*-tolu­aldehyde (2.4 mmol), β-naphthol (2 mmol), acetamide (2.4 mmol) in the presence of a catalytic amount of phenyl­boronic acid (1.5 mmol) was heated at 393 K without solvent for 7 h (the reaction was monitored by TLC). After completion of the reaction, the solid mixture was allowed to warm to room temperature, then 5 ml of 96% ethanol was added while maintaining stirring for 10 min. The solid was filtered, washed with cold 96% EtOH, dried and recrystallized from ethanol.

IR (KBr): ν (cm^−1^) 3405, 2921, 2358, 1627, 1508, 1265, 1065, 748, 686, 623. ^1^H NMR (DMSO-*d*_6_, 250 MHz): δ (ppm) 9.98 (*s*, 1H, –CONH), 8.28 (*d*, *J* = 8.7 Hz, 1H), 7.97 (*d*, *J* = 7.7 Hz, 1H), 7.74 (*d*, *J* = 8.0 Hz, 1H), 7.68 (*d*, *J* = 8.8 Hz, 1H), 7.40–6.92 (*m*, 7H), 2.22 (*s*, 3H, C_Ar_—CH_3_), 2.02 (*s*, 3H, CO—CH_3_). ^13^C NMR (DMSO-*d*_6_, 62.5 MHz): δ (ppm) 169.4, 153.1, 142.2, 137.0, 132.4, 129.0, 128.4, 127.7, 126.8, 126.6, 126.4, 123.2, 122.7, 122.4, 118.7, 118.6, 48.3, 22.9, 21.2.

## Refinement   

Crystal data, data collection and structure refinement details are summarized in Table 3 ▸. The hydroxyl H atoms were located in difference-Fourier maps but introduced in calculated positions and treated as riding: O—H = 0.82 Å, with *U*_iso_(H) = 1.5*U*_eq_(O). All other H atoms were positioned geometrically and refined as riding: N—H = 0.86, C—H = 0.93–0.96 Å with *U*_iso_(H) = 1.5U_eq_(*C*-meth­yl) and 1.2*U*_eq_(C,N) for other H atoms.

## Supplementary Material

Crystal structure: contains datablock(s) global, I. DOI: 10.1107/S2056989018008423/xu5928sup1.cif

Structure factors: contains datablock(s) I. DOI: 10.1107/S2056989018008423/xu5928Isup2.hkl

Supporting information file. DOI: 10.1107/S2056989018008423/xu5928Isup3.cml

CCDC reference: 1848011

Additional supporting information:  crystallographic information; 3D view; checkCIF report

## Figures and Tables

**Table 1:** Hydrogen-bond geometry (Å, °)

*D*—H⋯*A*	*D*—H	H⋯*A*	*D*⋯*A*	*D*—H⋯*A*
N1—H1*A*⋯O1	0.86	2.18	2.7424 (14)	123
N21—H21*A*⋯O21	0.86	2.35	2.8254 (15)	115
O1—H1⋯O2^i^	0.82	1.87	2.6298 (14)	153
O21—H21⋯O22^ii^	0.82	1.90	2.7111 (15)	169
C2—H2⋯O1^iii^	0.93	2.56	3.358 (2)	145
C13—H13⋯O2^i^	0.93	2.57	3.191 (2)	124

Symmetry codes: (i) 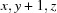; (ii) 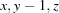; (iii) 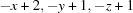.

**Table 2:** Summary of short inter­atomic contacts (Å)

Contact	Distance	Symmetry operation
C3⋯H10*A*	2.885	−*x* + 2, −*y* + 1, −*z* + 1
O1⋯O2	2.6298 (14)	*x*, *y* + 1, *z*
C2⋯H10*A*	2.80	−*x* + 2, −*y* + 1, −*z* + 1
O21⋯O22	2.7111 (15)	*x*, *y* − 1, *z*
O21⋯H22	2. 63	−*x* + 1, −*y*, −*z* + 1

**Table 3:** Experimental details

Crystal data	
Chemical formula	C_20_H_19_NO_2_
*M*_r_	305.36
Crystal system, space group	Monoclinic, *P*2_1_/*c*
Temperature (K)	293
*a*, *b*, *c* (Å)	24.3079 (16), 7.5677 (4), 18.4555 (14)
β (°)	110.024 (8)
*V* (Å^3^)	3189.7 (4)
*Z*	8
Radiation type	Mo *K*α
μ (mm^−1^)	0.08
Crystal size (mm)	0.26 × 0.13 × 0.09

Data collection	
Diffractometer	Agilent Technologies Xcalibur Eos
Absorption correction	Multi-scan (*CrysAlis PRO*; Agilent, 2014 ▸)
*T*_min_, *T*_max_	0.907, 1.000
No. of measured, independent and observed [*I* > 2σ(*I*)] reflections	23278, 10300, 6594
*R*_int_	0.025
(sin θ/λ)_max_ (Å^−1^)	0.756

Refinement	
*R*[*F* ^2^ > 2σ(*F* ^2^)], *wR*(*F* ^2^), *S*	0.056, 0.159, 1.03
No. of reflections	10300
No. of parameters	415
H-atom treatment	H-atom parameters constrained
Δρ_max_, Δρ_min_ (e Å^−3^)	0.29, −0.20

Computer programs: *CrysAlis PRO* (Agilent, 2014 ▸), *SIR92* (Altomare *et al.*, 1994 ▸), *SHELXL2013* (Sheldrick, 2015 ▸), *PLATON* (Spek, 2009 ▸), *Mercury* (Macrae *et al.*, 2008 ▸), *SHELXL2018* (Sheldrick, 2015 ▸) and *publCIF* (Westrip, 2010 ▸).

## supplementary crystallographic information

### Crystal data

**Table:**

C_20_H_19_NO_2_	*F*(000) = 1296
*M**_r_* = 305.36	*D*_x_ = 1.272 Mg m^−^^3^
Monoclinic, *P*2_1_/*c*	Mo *K*α radiation, λ = 0.7107 Å
*a* = 24.3079 (16) Å	Cell parameters from 5580 reflections
*b* = 7.5677 (4) Å	θ = 3.5–32.3°
*c* = 18.4555 (14) Å	µ = 0.08 mm^−^^1^
β = 110.024 (8)°	*T* = 293 K
*V* = 3189.7 (4) Å^3^	Needle, colorlese
*Z* = 8	0.26 × 0.13 × 0.09 mm

### Data collection

**Table:**

Agilent Technologies Xcalibur Eos diffractometer	10300 independent reflections
Radiation source: Enhance (Mo) X-ray Source	6594 reflections with *I* > 2σ(*I*)
Detector resolution: 8.0226 pixels mm^-1^	*R*_int_ = 0.025
ω scans	θ_max_ = 32.5°, θ_min_ = 3.2°
Absorption correction: multi-scan (CrysAlis PRO; Agilent, 2014)	*h* = −36→33
*T*_min_ = 0.907, *T*_max_ = 1.000	*k* = −10→10
23278 measured reflections	*l* = −12→27

### Refinement

**Table:**

Refinement on *F*^2^	Primary atom site location: structure-invariant direct methods
Least-squares matrix: full	Secondary atom site location: difference Fourier map
*R*[*F*^2^ > 2σ(*F*^2^)] = 0.056	Hydrogen site location: inferred from neighbouring sites
*wR*(*F*^2^) = 0.159	H-atom parameters constrained
*S* = 1.03	*w* = 1/[σ^2^(*F*_o_^2^) + (0.0676*P*)^2^ + 0.417*P*] where *P* = (*F*_o_^2^ + 2*F*_c_^2^)/3
10300 reflections	(Δ/σ)_max_ < 0.001
415 parameters	Δρ_max_ = 0.29 e Å^−^^3^
0 restraints	Δρ_min_ = −0.20 e Å^−^^3^

### Special details

**Table:**

Geometry. All esds (except the esd in the dihedral angle between two l.s. planes) are estimated using the full covariance matrix. The cell esds are taken into account individually in the estimation of esds in distances, angles and torsion angles; correlations between esds in cell parameters are only used when they are defined by crystal symmetry. An approximate (isotropic) treatment of cell esds is used for estimating esds involving l.s. planes.

### Fractional atomic coordinates and isotropic or equivalent isotropic displacement parameters (Å^2^)

**Table:**

	*x*	*y*	*z*	*U*_iso_*/*U*_eq_	
O1	0.92362 (5)	0.71780 (12)	0.51690 (7)	0.0382 (3)	
H1	0.9195	0.8173	0.4982	0.057*	
O21	0.56936 (5)	−0.16567 (13)	0.45919 (8)	0.0464 (3)	
H21	0.5613	−0.2681	0.4451	0.070*	
N1	0.92900 (5)	0.35840 (14)	0.50150 (7)	0.0306 (2)	
H1A	0.9321	0.4517	0.4766	0.037*	
N21	0.53975 (5)	0.19657 (14)	0.45137 (7)	0.0306 (2)	
H21A	0.5184	0.1032	0.4443	0.037*	
C21	0.61345 (6)	0.12477 (17)	0.57884 (8)	0.0291 (3)	
C216	0.67823 (6)	0.12927 (18)	0.42822 (8)	0.0305 (3)	
O22	0.54173 (6)	0.48795 (14)	0.43134 (9)	0.0563 (4)	
C27	0.60251 (6)	0.17987 (16)	0.49558 (8)	0.0278 (3)	
H27	0.6192	0.2984	0.4978	0.033*	
C7	0.92286 (6)	0.37875 (16)	0.57749 (8)	0.0287 (3)	
H7	0.9068	0.2665	0.5877	0.034*	
C217	0.70322 (7)	0.3003 (2)	0.44704 (9)	0.0376 (3)	
H217	0.6891	0.3759	0.4764	0.045*	
C22	0.57640 (7)	0.18155 (19)	0.61706 (9)	0.0353 (3)	
H22	0.5428	0.2445	0.5898	0.042*	
C15	0.78567 (6)	0.60938 (19)	0.59489 (9)	0.0348 (3)	
C9	0.92993 (6)	0.20029 (17)	0.46945 (9)	0.0339 (3)	
C2	1.03272 (6)	0.33243 (19)	0.63590 (9)	0.0350 (3)	
H2	1.0314	0.2807	0.5896	0.042*	
C16	0.83131 (6)	0.48354 (18)	0.60542 (8)	0.0315 (3)	
O2	0.92749 (6)	0.06341 (14)	0.50457 (8)	0.0561 (4)	
C211	0.63209 (6)	0.06506 (17)	0.45251 (8)	0.0293 (3)	
C12	0.87790 (6)	0.68069 (17)	0.54142 (8)	0.0295 (3)	
C1	0.98150 (6)	0.40135 (17)	0.64271 (8)	0.0306 (3)	
C212	0.61263 (7)	−0.10654 (18)	0.43421 (9)	0.0355 (3)	
C11	0.87709 (6)	0.51816 (17)	0.57498 (8)	0.0282 (3)	
C17	0.82953 (7)	0.3286 (2)	0.64798 (11)	0.0446 (4)	
H17	0.8594	0.2455	0.6573	0.053*	
C24	0.63861 (8)	0.0530 (2)	0.73514 (10)	0.0470 (4)	
H24	0.6475	0.0298	0.7874	0.056*	
C14	0.78716 (7)	0.7681 (2)	0.55555 (10)	0.0390 (3)	
H14	0.7568	0.8490	0.5467	0.047*	
C13	0.83230 (7)	0.80449 (18)	0.53048 (9)	0.0358 (3)	
H13	0.8332	0.9113	0.5060	0.043*	
C214	0.67914 (8)	−0.1549 (2)	0.36602 (12)	0.0513 (4)	
H214	0.6940	−0.2280	0.3366	0.062*	
C26	0.66307 (7)	0.0296 (2)	0.62030 (10)	0.0402 (3)	
H26	0.6883	−0.0107	0.5958	0.048*	
C6	0.98401 (7)	0.4762 (2)	0.71238 (10)	0.0410 (4)	
H6	0.9504	0.5239	0.7178	0.049*	
C25	0.67531 (8)	−0.0057 (2)	0.69808 (11)	0.0485 (4)	
H25	0.7087	−0.0698	0.7252	0.058*	
C4	1.08714 (8)	0.4130 (2)	0.76614 (11)	0.0468 (4)	
H4	1.1222	0.4173	0.8076	0.056*	
C3	1.08604 (7)	0.3394 (2)	0.69714 (10)	0.0412 (4)	
C219	0.77018 (7)	0.2456 (3)	0.37928 (11)	0.0519 (4)	
H219	0.8004	0.2847	0.3632	0.062*	
C29	0.51402 (6)	0.34835 (18)	0.42162 (9)	0.0336 (3)	
C10	0.93414 (8)	0.1965 (2)	0.39073 (11)	0.0473 (4)	
H10A	0.9355	0.3153	0.3732	0.071*	
H10B	0.9005	0.1371	0.3560	0.071*	
H10C	0.9690	0.1347	0.3924	0.071*	
C20	0.73944 (7)	0.5718 (2)	0.62321 (10)	0.0449 (4)	
H20	0.7090	0.6523	0.6147	0.054*	
C215	0.70185 (7)	0.0178 (2)	0.38405 (9)	0.0388 (3)	
C220	0.74777 (8)	0.0807 (3)	0.36040 (11)	0.0512 (4)	
H220	0.7630	0.0077	0.3313	0.061*	
C23	0.58816 (8)	0.1468 (2)	0.69530 (10)	0.0433 (4)	
C213	0.63629 (8)	−0.2162 (2)	0.39051 (11)	0.0484 (4)	
H213	0.6224	−0.3309	0.3785	0.058*	
C210	0.44988 (7)	0.3386 (2)	0.37528 (12)	0.0503 (4)	
H210A	0.4366	0.2190	0.3747	0.076*	
H210B	0.4436	0.3766	0.3234	0.076*	
H210C	0.4285	0.4139	0.3981	0.076*	
C218	0.74773 (7)	0.3560 (2)	0.42277 (10)	0.0461 (4)	
H218	0.7631	0.4689	0.4355	0.055*	
C18	0.78482 (8)	0.2994 (3)	0.67542 (12)	0.0529 (5)	
H18	0.7849	0.1970	0.7033	0.063*	
C19	0.73898 (8)	0.4201 (3)	0.66252 (11)	0.0507 (4)	
H19	0.7084	0.3969	0.6807	0.061*	
C5	1.03648 (8)	0.4802 (2)	0.77408 (11)	0.0492 (4)	
H5	1.0376	0.5283	0.8209	0.059*	
C28	0.54747 (12)	0.2098 (4)	0.73576 (14)	0.0749 (7)	
H28A	0.5624	0.1741	0.7889	0.112*	
H28B	0.5094	0.1591	0.7114	0.112*	
H28C	0.5447	0.3363	0.7328	0.112*	
C8	1.14143 (8)	0.2672 (3)	0.68826 (14)	0.0649 (6)	
H8A	1.1327	0.2212	0.6371	0.097*	
H8B	1.1569	0.1745	0.7251	0.097*	
H8C	1.1698	0.3602	0.6969	0.097*	

### Atomic displacement parameters (Å^2^)

**Table:**

	*U*^11^	*U*^22^	*U*^33^	*U*^12^	*U*^13^	*U*^23^
O1	0.0442 (6)	0.0212 (4)	0.0593 (7)	0.0018 (4)	0.0308 (5)	0.0060 (4)
O21	0.0532 (7)	0.0250 (5)	0.0713 (8)	−0.0102 (4)	0.0348 (6)	−0.0091 (5)
N1	0.0364 (6)	0.0199 (5)	0.0361 (6)	−0.0002 (4)	0.0131 (5)	0.0017 (4)
N21	0.0328 (6)	0.0218 (5)	0.0375 (7)	−0.0029 (4)	0.0125 (5)	0.0007 (4)
C21	0.0323 (7)	0.0236 (6)	0.0326 (7)	−0.0022 (5)	0.0129 (6)	−0.0008 (5)
C216	0.0306 (6)	0.0346 (7)	0.0262 (6)	0.0002 (5)	0.0094 (5)	0.0007 (5)
O22	0.0550 (7)	0.0217 (5)	0.0844 (10)	−0.0031 (5)	0.0137 (7)	0.0039 (5)
C27	0.0307 (6)	0.0214 (5)	0.0334 (7)	−0.0018 (5)	0.0138 (5)	−0.0016 (5)
C7	0.0304 (6)	0.0216 (6)	0.0357 (7)	0.0006 (5)	0.0134 (6)	0.0036 (5)
C217	0.0391 (8)	0.0425 (8)	0.0335 (8)	−0.0097 (6)	0.0154 (6)	−0.0029 (6)
C22	0.0365 (7)	0.0356 (7)	0.0364 (8)	0.0049 (6)	0.0160 (6)	0.0011 (6)
C15	0.0312 (7)	0.0379 (7)	0.0366 (8)	−0.0006 (6)	0.0135 (6)	−0.0049 (6)
C9	0.0317 (7)	0.0235 (6)	0.0472 (9)	−0.0013 (5)	0.0144 (6)	−0.0036 (6)
C2	0.0346 (7)	0.0344 (7)	0.0366 (8)	0.0015 (6)	0.0127 (6)	0.0013 (6)
C16	0.0301 (6)	0.0311 (7)	0.0337 (7)	−0.0021 (5)	0.0117 (6)	0.0014 (5)
O2	0.0834 (9)	0.0205 (5)	0.0766 (9)	−0.0016 (5)	0.0432 (8)	0.0008 (5)
C211	0.0317 (7)	0.0276 (6)	0.0304 (7)	−0.0001 (5)	0.0129 (5)	−0.0014 (5)
C12	0.0318 (6)	0.0246 (6)	0.0342 (7)	0.0002 (5)	0.0141 (6)	0.0009 (5)
C1	0.0345 (7)	0.0213 (6)	0.0361 (7)	0.0011 (5)	0.0121 (6)	0.0036 (5)
C212	0.0387 (7)	0.0274 (7)	0.0428 (8)	−0.0015 (5)	0.0167 (6)	−0.0048 (6)
C11	0.0276 (6)	0.0248 (6)	0.0326 (7)	0.0012 (5)	0.0110 (5)	0.0016 (5)
C17	0.0440 (9)	0.0422 (8)	0.0508 (10)	−0.0002 (7)	0.0205 (8)	0.0125 (7)
C24	0.0557 (10)	0.0484 (9)	0.0350 (8)	−0.0033 (8)	0.0128 (8)	0.0063 (7)
C14	0.0343 (7)	0.0360 (7)	0.0473 (9)	0.0087 (6)	0.0149 (7)	−0.0007 (6)
C13	0.0417 (8)	0.0249 (6)	0.0418 (8)	0.0066 (5)	0.0156 (7)	0.0052 (6)
C214	0.0584 (11)	0.0471 (9)	0.0564 (11)	0.0065 (8)	0.0299 (9)	−0.0146 (8)
C26	0.0376 (8)	0.0408 (8)	0.0440 (9)	0.0069 (6)	0.0162 (7)	0.0040 (7)
C6	0.0445 (8)	0.0353 (8)	0.0441 (9)	0.0043 (6)	0.0163 (7)	−0.0051 (6)
C25	0.0446 (9)	0.0497 (9)	0.0456 (10)	0.0085 (7)	0.0080 (8)	0.0122 (8)
C4	0.0446 (9)	0.0390 (8)	0.0450 (10)	−0.0014 (7)	−0.0001 (7)	−0.0004 (7)
C3	0.0360 (8)	0.0383 (8)	0.0456 (9)	0.0024 (6)	0.0092 (7)	0.0052 (7)
C219	0.0357 (8)	0.0759 (13)	0.0489 (10)	−0.0040 (8)	0.0207 (7)	0.0102 (9)
C29	0.0386 (7)	0.0248 (6)	0.0393 (8)	0.0007 (5)	0.0159 (6)	0.0005 (5)
C10	0.0535 (10)	0.0407 (9)	0.0503 (10)	−0.0039 (7)	0.0213 (8)	−0.0123 (7)
C20	0.0373 (8)	0.0522 (9)	0.0504 (10)	−0.0037 (7)	0.0218 (7)	−0.0109 (8)
C215	0.0369 (7)	0.0463 (8)	0.0363 (8)	0.0065 (6)	0.0165 (6)	0.0002 (6)
C220	0.0455 (9)	0.0694 (12)	0.0473 (10)	0.0113 (8)	0.0269 (8)	0.0031 (9)
C23	0.0516 (9)	0.0458 (9)	0.0377 (8)	0.0003 (7)	0.0222 (7)	0.0003 (7)
C213	0.0562 (10)	0.0323 (8)	0.0625 (12)	−0.0017 (7)	0.0275 (9)	−0.0151 (7)
C210	0.0430 (9)	0.0403 (9)	0.0606 (12)	0.0038 (7)	0.0086 (8)	0.0067 (8)
C218	0.0402 (8)	0.0580 (10)	0.0401 (9)	−0.0148 (7)	0.0136 (7)	0.0016 (8)
C18	0.0542 (10)	0.0539 (10)	0.0581 (12)	−0.0071 (8)	0.0289 (9)	0.0143 (9)
C19	0.0468 (9)	0.0613 (11)	0.0540 (11)	−0.0136 (8)	0.0303 (8)	−0.0072 (9)
C5	0.0584 (11)	0.0426 (9)	0.0412 (9)	0.0005 (7)	0.0102 (8)	−0.0108 (7)
C28	0.0897 (16)	0.0980 (17)	0.0534 (13)	0.0242 (13)	0.0456 (12)	0.0081 (12)
C8	0.0353 (9)	0.0847 (15)	0.0676 (14)	0.0108 (9)	0.0084 (9)	−0.0033 (11)

### Geometric parameters (Å, º)

**Table:**

O1—C12	1.3651 (17)	C24—C23	1.390 (2)
O1—H1	0.8200	C24—H24	0.9300
O21—C212	1.3605 (19)	C14—C13	1.357 (2)
O21—H21	0.8200	C14—H14	0.9300
N1—C9	1.3384 (17)	C13—H13	0.9300
N1—C7	1.4683 (19)	C214—C213	1.351 (3)
N1—H1A	0.8600	C214—C215	1.413 (2)
N21—C29	1.3329 (18)	C214—H214	0.9300
N21—C27	1.4685 (18)	C26—C25	1.389 (2)
N21—H21A	0.8600	C26—H26	0.9300
C21—C26	1.388 (2)	C6—C5	1.389 (2)
C21—C22	1.389 (2)	C6—H6	0.9300
C21—C27	1.525 (2)	C25—H25	0.9300
C216—C217	1.422 (2)	C4—C3	1.382 (3)
C216—C215	1.424 (2)	C4—C5	1.386 (3)
C216—C211	1.4281 (19)	C4—H4	0.9300
O22—C29	1.2324 (17)	C3—C8	1.513 (2)
C27—C211	1.5149 (19)	C219—C220	1.359 (3)
C27—H27	0.9800	C219—C218	1.393 (3)
C7—C11	1.5224 (18)	C219—H219	0.9300
C7—C1	1.528 (2)	C29—C210	1.502 (2)
C7—H7	0.9800	C10—H10A	0.9600
C217—C218	1.371 (2)	C10—H10B	0.9600
C217—H217	0.9300	C10—H10C	0.9600
C22—C23	1.398 (2)	C20—C19	1.360 (3)
C22—H22	0.9300	C20—H20	0.9300
C15—C14	1.410 (2)	C215—C220	1.413 (2)
C15—C20	1.421 (2)	C220—H220	0.9300
C15—C16	1.424 (2)	C23—C28	1.506 (3)
C9—O2	1.2341 (18)	C213—H213	0.9300
C9—C10	1.491 (2)	C210—H210A	0.9600
C2—C1	1.395 (2)	C210—H210B	0.9600
C2—C3	1.399 (2)	C210—H210C	0.9600
C2—H2	0.9300	C218—H218	0.9300
C16—C17	1.420 (2)	C18—C19	1.397 (3)
C16—C11	1.4323 (19)	C18—H18	0.9300
C211—C212	1.3838 (19)	C19—H19	0.9300
C12—C11	1.3803 (18)	C5—H5	0.9300
C12—C13	1.4120 (19)	C28—H28A	0.9600
C1—C6	1.387 (2)	C28—H28B	0.9600
C212—C213	1.410 (2)	C28—H28C	0.9600
C17—C18	1.365 (2)	C8—H8A	0.9600
C17—H17	0.9300	C8—H8B	0.9600
C24—C25	1.371 (3)	C8—H8C	0.9600
			
C12—O1—H1	109.5	C215—C214—H214	119.3
C212—O21—H21	109.5	C21—C26—C25	120.53 (15)
C9—N1—C7	122.58 (12)	C21—C26—H26	119.7
C9—N1—H1A	118.7	C25—C26—H26	119.7
C7—N1—H1A	118.7	C1—C6—C5	120.36 (15)
C29—N21—C27	123.69 (11)	C1—C6—H6	119.8
C29—N21—H21A	118.2	C5—C6—H6	119.8
C27—N21—H21A	118.2	C24—C25—C26	120.51 (15)
C26—C21—C22	118.21 (14)	C24—C25—H25	119.7
C26—C21—C27	121.07 (13)	C26—C25—H25	119.7
C22—C21—C27	120.50 (12)	C3—C4—C5	120.53 (16)
C217—C216—C215	117.21 (13)	C3—C4—H4	119.7
C217—C216—C211	123.30 (13)	C5—C4—H4	119.7
C215—C216—C211	119.48 (13)	C4—C3—C2	118.70 (15)
N21—C27—C211	110.32 (11)	C4—C3—C8	120.54 (16)
N21—C27—C21	111.90 (11)	C2—C3—C8	120.76 (17)
C211—C27—C21	114.93 (11)	C220—C219—C218	119.79 (16)
N21—C27—H27	106.4	C220—C219—H219	120.1
C211—C27—H27	106.4	C218—C219—H219	120.1
C21—C27—H27	106.4	O22—C29—N21	121.52 (14)
N1—C7—C11	110.84 (11)	O22—C29—C210	122.30 (13)
N1—C7—C1	113.06 (11)	N21—C29—C210	116.19 (12)
C11—C7—C1	114.91 (11)	C9—C10—H10A	109.5
N1—C7—H7	105.7	C9—C10—H10B	109.5
C11—C7—H7	105.7	H10A—C10—H10B	109.5
C1—C7—H7	105.7	C9—C10—H10C	109.5
C218—C217—C216	121.25 (15)	H10A—C10—H10C	109.5
C218—C217—H217	119.4	H10B—C10—H10C	109.5
C216—C217—H217	119.4	C19—C20—C15	121.13 (16)
C21—C22—C23	121.86 (14)	C19—C20—H20	119.4
C21—C22—H22	119.1	C15—C20—H20	119.4
C23—C22—H22	119.1	C220—C215—C214	121.67 (15)
C14—C15—C20	121.51 (14)	C220—C215—C216	119.56 (15)
C14—C15—C16	118.93 (13)	C214—C215—C216	118.76 (15)
C20—C15—C16	119.55 (14)	C219—C220—C215	121.34 (17)
O2—C9—N1	120.47 (15)	C219—C220—H220	119.3
O2—C9—C10	121.82 (14)	C215—C220—H220	119.3
N1—C9—C10	117.71 (13)	C24—C23—C22	118.29 (15)
C1—C2—C3	121.46 (15)	C24—C23—C28	120.65 (17)
C1—C2—H2	119.3	C22—C23—C28	121.06 (16)
C3—C2—H2	119.3	C214—C213—C212	120.09 (15)
C17—C16—C15	117.26 (14)	C214—C213—H213	120.0
C17—C16—C11	122.87 (13)	C212—C213—H213	120.0
C15—C16—C11	119.85 (13)	C29—C210—H210A	109.5
C212—C211—C216	118.84 (13)	C29—C210—H210B	109.5
C212—C211—C27	118.84 (12)	H210A—C210—H210B	109.5
C216—C211—C27	122.30 (12)	C29—C210—H210C	109.5
O1—C12—C11	118.06 (12)	H210A—C210—H210C	109.5
O1—C12—C13	120.29 (12)	H210B—C210—H210C	109.5
C11—C12—C13	121.63 (13)	C217—C218—C219	120.84 (16)
C6—C1—C2	118.62 (14)	C217—C218—H218	119.6
C6—C1—C7	120.59 (13)	C219—C218—H218	119.6
C2—C1—C7	120.53 (13)	C17—C18—C19	121.43 (17)
O21—C212—C211	117.73 (13)	C17—C18—H18	119.3
O21—C212—C213	120.96 (13)	C19—C18—H18	119.3
C211—C212—C213	121.31 (14)	C20—C19—C18	119.41 (16)
C12—C11—C16	118.12 (12)	C20—C19—H19	120.3
C12—C11—C7	120.58 (12)	C18—C19—H19	120.3
C16—C11—C7	121.29 (11)	C4—C5—C6	120.32 (16)
C18—C17—C16	121.15 (16)	C4—C5—H5	119.8
C18—C17—H17	119.4	C6—C5—H5	119.8
C16—C17—H17	119.4	C23—C28—H28A	109.5
C25—C24—C23	120.58 (16)	C23—C28—H28B	109.5
C25—C24—H24	119.7	H28A—C28—H28B	109.5
C23—C24—H24	119.7	C23—C28—H28C	109.5
C13—C14—C15	120.97 (13)	H28A—C28—H28C	109.5
C13—C14—H14	119.5	H28B—C28—H28C	109.5
C15—C14—H14	119.5	C3—C8—H8A	109.5
C14—C13—C12	120.22 (13)	C3—C8—H8B	109.5
C14—C13—H13	119.9	H8A—C8—H8B	109.5
C12—C13—H13	119.9	C3—C8—H8C	109.5
C213—C214—C215	121.49 (15)	H8A—C8—H8C	109.5
C213—C214—H214	119.3	H8B—C8—H8C	109.5
			
C29—N21—C27—C211	−114.66 (14)	N1—C7—C11—C16	130.38 (13)
C29—N21—C27—C21	116.05 (14)	C1—C7—C11—C16	−99.91 (15)
C26—C21—C27—N21	151.70 (13)	C15—C16—C17—C18	2.0 (3)
C22—C21—C27—N21	−33.75 (17)	C11—C16—C17—C18	−179.34 (16)
C26—C21—C27—C211	24.87 (18)	C20—C15—C14—C13	178.32 (15)
C22—C21—C27—C211	−160.59 (12)	C16—C15—C14—C13	−2.5 (2)
C9—N1—C7—C11	−133.02 (13)	C15—C14—C13—C12	1.9 (2)
C9—N1—C7—C1	96.29 (15)	O1—C12—C13—C14	−179.37 (14)
C215—C216—C217—C218	0.8 (2)	C11—C12—C13—C14	2.4 (2)
C211—C216—C217—C218	179.63 (15)	C22—C21—C26—C25	−0.6 (2)
C26—C21—C22—C23	0.6 (2)	C27—C21—C26—C25	174.04 (14)
C27—C21—C22—C23	−174.10 (14)	C2—C1—C6—C5	−0.7 (2)
C7—N1—C9—O2	−2.4 (2)	C7—C1—C6—C5	173.47 (14)
C7—N1—C9—C10	177.78 (13)	C23—C24—C25—C26	0.9 (3)
C14—C15—C16—C17	177.55 (15)	C21—C26—C25—C24	−0.1 (3)
C20—C15—C16—C17	−3.2 (2)	C5—C4—C3—C2	−0.8 (2)
C14—C15—C16—C11	−1.1 (2)	C5—C4—C3—C8	179.53 (18)
C20—C15—C16—C11	178.11 (14)	C1—C2—C3—C4	1.5 (2)
C217—C216—C211—C212	−177.01 (14)	C1—C2—C3—C8	−178.80 (16)
C215—C216—C211—C212	1.8 (2)	C27—N21—C29—O22	−1.9 (2)
C217—C216—C211—C27	4.8 (2)	C27—N21—C29—C210	177.50 (14)
C215—C216—C211—C27	−176.35 (13)	C14—C15—C20—C19	−178.58 (17)
N21—C27—C211—C212	−58.80 (17)	C16—C15—C20—C19	2.2 (2)
C21—C27—C211—C212	68.83 (17)	C213—C214—C215—C220	178.20 (18)
N21—C27—C211—C216	119.38 (14)	C213—C214—C215—C216	−0.8 (3)
C21—C27—C211—C216	−112.99 (14)	C217—C216—C215—C220	−0.7 (2)
C3—C2—C1—C6	−0.8 (2)	C211—C216—C215—C220	−179.58 (14)
C3—C2—C1—C7	−174.92 (13)	C217—C216—C215—C214	178.34 (15)
N1—C7—C1—C6	159.58 (13)	C211—C216—C215—C214	−0.6 (2)
C11—C7—C1—C6	30.96 (18)	C218—C219—C220—C215	0.0 (3)
N1—C7—C1—C2	−26.40 (17)	C214—C215—C220—C219	−178.66 (18)
C11—C7—C1—C2	−155.01 (13)	C216—C215—C220—C219	0.3 (3)
C216—C211—C212—O21	178.84 (13)	C25—C24—C23—C22	−0.9 (3)
C27—C211—C212—O21	−2.9 (2)	C25—C24—C23—C28	179.38 (19)
C216—C211—C212—C213	−1.8 (2)	C21—C22—C23—C24	0.1 (2)
C27—C211—C212—C213	176.44 (15)	C21—C22—C23—C28	179.89 (18)
O1—C12—C11—C16	175.87 (12)	C215—C214—C213—C212	0.9 (3)
C13—C12—C11—C16	−5.8 (2)	O21—C212—C213—C214	179.81 (17)
O1—C12—C11—C7	−5.6 (2)	C211—C212—C213—C214	0.5 (3)
C13—C12—C11—C7	172.67 (13)	C216—C217—C218—C219	−0.5 (3)
C17—C16—C11—C12	−173.44 (15)	C220—C219—C218—C217	0.1 (3)
C15—C16—C11—C12	5.1 (2)	C16—C17—C18—C19	0.3 (3)
C17—C16—C11—C7	8.1 (2)	C15—C20—C19—C18	0.2 (3)
C15—C16—C11—C7	−173.32 (13)	C17—C18—C19—C20	−1.4 (3)
N1—C7—C11—C12	−48.06 (17)	C3—C4—C5—C6	−0.7 (3)
C1—C7—C11—C12	81.66 (17)	C1—C6—C5—C4	1.4 (3)

### Hydrogen-bond geometry (Å, º)

**Table:**

*D*—H···*A*	*D*—H	H···*A*	*D*···*A*	*D*—H···*A*
N1—H1*A*···O1	0.86	2.18	2.7424 (14)	123
N21—H21*A*···O21	0.86	2.35	2.8254 (15)	115
O1—H1···O2^i^	0.82	1.87	2.6298 (14)	153
O21—H21···O22^ii^	0.82	1.90	2.7111 (15)	169
C2—H2···O1^iii^	0.93	2.56	3.358 (2)	145
C13—H13···O2^i^	0.93	2.57	3.191 (2)	124

Symmetry codes: (i) *x*, *y*+1, *z*; (ii) *x*, *y*−1, *z*; (iii) −*x*+2, −*y*+1, −*z*+1.
