# Crystal structure and Hirshfeld surface analysis of *N*-[(2-hy­droxy­naphthalen-1-yl)(3-methyl­phen­yl)meth­yl]acetamide. Corrigendum

**DOI:** 10.1107/S2056989022007277

**Published:** 2022-07-22

**Authors:** Khawla Boudebbous, Wissame Zemamouche, Abdelmadjid Debache, Noudjoud Hamdouni, Ali Boudjada

**Affiliations:** a Laboratoire de Synthèse de Molécules, d’Intérêts Biologiques, Département de Chimie, Université Mentouri-Constantine, 25000 Constantine, Algeria; b Laboratoire de Cristallographie, Département de Physique, Université Mentouri-Constantine, 25000 Constantine, Algeria

## Abstract

In the paper by Boudebbous *et al.* [ *Acta Cryst.* (2018), E**74**, 1002–1005], there is an error in the name of the second author.

## Abstract 2

Corrigendum to *Acta Cryst.* (2018), E**74**, 1002–1005.

The name of the second author in the paper by Boudebbous *et al.* (2018 ▸) is incorrect and should be ‘Wissame Zemamouche’ as given above.
